# Development and Validation of an Instrument for Measuring the Quality of Teamwork in Teaching Teams in Postgraduate Medical Training (TeamQ)

**DOI:** 10.1371/journal.pone.0112805

**Published:** 2014-11-13

**Authors:** Irene A. Slootweg, Kiki M. J. M. H. Lombarts, Benjamin C. M. Boerebach, Maas Jan Heineman, Albert J. J. A. Scherpbier, Cees P. M. van der Vleuten

**Affiliations:** 1 Professional Performance Research group, Center of Expertise in Evidence-based Education, Academic Medical Center, University of Amsterdam, Amsterdam, the Netherlands; 2 Department of Educational Development and Research, University of Maastricht, Maastricht, the Netherlands; 3 Faculty of Health, Medicine and Life Sciences, University of Maastricht, Maastricht, the Netherlands; Georgetown University Medical Center, United States of America

## Abstract

**Background:**

Teamwork between clinical teachers is a challenge in postgraduate medical training. Although there are several instruments available for measuring teamwork in health care, none of them are appropriate for teaching teams. The aim of this study is to develop an instrument (TeamQ) for measuring teamwork, to investigate its psychometric properties and to explore how clinical teachers assess their teamwork.

**Method:**

To select the items to be included in the TeamQ questionnaire, we conducted a content validation in 2011, using a Delphi procedure in which 40 experts were invited. Next, for pilot testing the preliminary tool, 1446 clinical teachers from 116 teaching teams were requested to complete the TeamQ questionnaire. For data analyses we used statistical strategies: principal component analysis, internal consistency reliability coefficient, and the number of evaluations needed to obtain reliable estimates. Lastly, the median TeamQ scores were calculated for teams to explore the levels of teamwork.

**Results:**

In total, 31 experts participated in the Delphi study. In total, 114 teams participated in the TeamQ pilot. The median team response was 7 evaluations per team. The principal component analysis revealed 11 factors; 8 were included. The reliability coefficients of the TeamQ scales ranged from 0.75 to 0.93. The generalizability analysis revealed that 5 to 7 evaluations were needed to obtain internal reliability coefficients of 0.70. In terms of teamwork, the clinical teachers scored *residents' empowerment* as the highest TeamQ scale and *feedback culture* as the area that would most benefit from improvement.

**Conclusions:**

This study provides initial evidence of the validity of an instrument for measuring teamwork in teaching teams. The high response rates and the low number of evaluations needed for reliably measuring teamwork indicate that TeamQ is feasible for use by teaching teams. Future research could explore the effectiveness of feedback on teamwork in follow up measurements.

## Introduction

Tackling the issue of teamwork is one of the challenges in reforming professional health education. [1] This also applies to teamwork for clinical teachers in postgraduate medical training. Recent studies report that clinical teachers are more aware of the necessity for teamwork in delivering high quality residency training. [2]–[4] In particular, they acknowledge the need to agree upon and commit to professional standards and common approaches to supervising and assessing residents, sharing educational tasks as well as assuring the quality and improvement of the training program. Teamwork is a well-researched phenomenon, where the focus is most commonly on three lines of research: the teamwork skills of individual team members, the team process and team results. [5]–[7] In view of the collective responsibility for team results, it is important that these three research lines on teamwork are addressed. [5], [8], [9] With regard to the first line of research, Burke presents a model for teamwork skills, including distinguishing knowledge, attitudes, traits and abilities. [10], [11] The second line of research, the team process, connects team members' individual teamwork skills with the team results. The team process is frequently considered to be a black box of teamwork, because it is unclear what really happens when a team member with the right teamwork skills does not achieve the right team results. [6] Denecker operationalized team process indicators for multi-disciplinary teams as follows: team relations, quality of team leadership, team communication, team/task reflexivity, team vision, task orientation, team mental model, belief that multidisciplinary patient care teams result in better outcomes. [7] Measuring team results, the third line of teamwork research is challenging, mainly because the results of teamwork are often unclear and can be different for individual team members. [5], [12], [13] Outcome indicators in health care teams include teams' perceived coordination of the care process, as well as team effectiveness, teams' perceived communication with patient and family, team satisfaction, teams' perceived follow−up of the care process and professional agreement on best practices. [14] The design of this study was based on the above described three lines of research on teamwork: teamwork skills, team processes and team results. More specifically, we wanted to identify criteria for measuring teamwork skills, team processes and team results in teaching teams. Insight in actual levels of teamwork, including the strength and weaknesses, is a necessary first step in the process of continuous QI, also known as Quality Improvement or PDCA cycles. [15], [16] After this first step, evaluation, followed by reflection and improvement actions, is possible in the context of achieving or maintaining effective teamwork. Even though many measurement instruments are available for evaluating teamwork in health care teams, [14] no particular instrument is specific enough for use in teaching teams in residency training. This study aims to develop and validate such an instrument and to explore how clinical teachers appraise their current levels of teamwork. More specifically, our research questions are: (i) to investigate whether teamwork in teaching teams in the context of residency training can be measured validly and reliably, and (ii) to explore how individual members of teaching teams evaluate their current levels of teamwork. To develop and validate an evaluation instrument (TeamQ), we used a mixed-methods approach based on a modified Delphi procedure, followed by psychometric analyses of the instrument.

## Methodology

### Setting

Postgraduate medical training in the Netherlands is organized in eight geographical regions, each of which is coordinated by one university medical center. In all regional affiliated hospitals, residents work alongside clinical teachers, who also act as their supervisors. Each program is coordinated by a local program director, who is responsible for the quality and delivery of the program in the workplace and the mutual performance of the clinical teachers. The clinical teacher, also named supervisor, is the medical doctor working with residents on a daily basis in the workplace, supervising and assessing the residents ‘medical activities, as well as teaching them professional knowledge, skills and attitudes. In most western health care systems, competency-based residency training has been introduced over the past decade. As a result, residents, in various settings, learn from a wide range of different situations under the supervision of multiple clinical teachers. This makes teamwork for supervising and assessing the residents necessary for clinical teachers. [17]

Waiver of ethical approval was provided by the Institutional Review Board of the Academic Medical Center of the University of Amsterdam, Amsterdam, The Netherlands. A waiver was provided because ethical approval for this study was not required under Dutch law.

### Method

This study uses a mixed methods approach. The quantitative statistical analysis reflected in the study indicates a post-positivistic approach, a paradigm based on the assumption that there is one truth, but it can never be truly observed. A more constructivistic approach, assuming multiple truths are constructed by and between people, is reflected during the Delphi procedure and is built on stressing the frequent discussion sessions within the research group and on the dialogue with the target group of clinical teachers. [18], [19] We answered the first research question, that is how to validly and reliably measure teamwork, by developing the TeamQ instrument during three consecutive phases. [20] The second research question, that is how clinical teachers assess their current levels of teamwork, was answered through the analysis of the available TeamQ data that also yielded the median scores per team.

#### 1. Selecting items with a Delphi procedure

We based the definition and first selection of the teamwork items on our previous study on teamwork for clinical teachers. [4] This focus group study revealed 7 preliminary teamwork themes, namely: the clinical teacher, the residents, the program director, the content, the structure, the feedback and the environment. We initially operationalized these themes into 86 teamwork items. (Table S1). Subsequently, we performed a modified Delphi procedure. A Delphi procedure is aimed at achieving consensus among experts in a systematic manner. [20], [21] In a modified Delphi procedure, the items are not generated by the expert group but – as in this study – are selected based on earlier research. [4], [21], [22] Forty experts from diverse professional backgrounds were invited to participate by telephone or email. In total, 10 program directors, 10 educationalists, 10 supervisors and 10 residents were purposefully selected through the network of the research group. The voluntary nature of participation was emphasized in the instruction email. From August to December 2011, the 86 items that were defined in the focus group study [4] were critically reviewed during the first round of the Delphi procedure. [20], [22]–[24] In the first round the experts rated the relevance of each item on a four-point scale, from irrelevant, to highly relevant. [21] We also asked the experts to give feedback on the formulation of the items and to indicate whether any particular dimensions of teamwork were underexposed. Each of the four expert groups was first analyzed separately and then combined at a later stage. The relevance of items was analyzed by calculating the mean relevance scores. These relevance scores were then plotted and inspected visually, both per expert subgroup and for all experts combined. Based on the visual inspection, items that showed consistently low relevance scores were excluded. We did not use one uniform cut-off value because of the heterogeneity between our expert subgroups. More specifically, some items were experienced as being very relevant by residents, but not by program directors and supervisors and some items about recently introduced/renewed regulations (that will soon become very relevant in practice) were perceived as very relevant by educationalists, but not yet by supervisors or residents. Averaging all items and checking them at a uniform cut-off value would have resulted in deletion of such items. All remaining items proceeded to the second Delphi round. In this round, the experts judged items clarity on a three-point scale (1 =  clear, 2 =  neutral and 3 =  not clear). In addition, they were asked to prioritize the items for measuring teamwork. After the second round, data were analyzed in the same way as the first round. The formulation, clarity and relevance of the items were discussed extensively in the research group. In addition, the prioritization of the various expert subgroups was included in the research group's final choice of items to be included in the TeamQ instrument. The online questionnaire was provided and answered in Dutch.

#### 2. Testing the TeamQ instrument

To test the TeamQ instrument in practice, an internet-based environment was developed to facilitate the data collection. From January 2012 to December 2013 the instrument was offered to teaching teams from multiple specialties and multiple teaching hospitals. We use the multiple specialties and the multiple teaching hospitals to achieve an inclusive and representative sample of teaching teams. In total 116 teaching teams (1446 clinical teachers) representing 34 hospitals were invited to complete the TeamQ instrument. Teaching teams were approached in person, by email or through telephone contact. Teams were actively recruited using the network of the research group. Teams that were already familiar with the professional performance online program (www.professionalperformanceonline.com), to which TeamQ was newly added, could also request use of the TeamQ in the pilot phase. Respondents were asked - in a self-reported performance assessment - to rate to what degree the situation presented in an item was valid for teamwork in their own teaching team. The measurement period lasted one month. The system was programmed to remind respondents to fill in the TeamQ questionnaire three times during this period. At the end of the measurement period, a single report summarizing the team results, was automatically generated and sent to all team members.

#### 3. Statistical analyses

We carried out various statistical analyses to explore the validity and reliability of the TeamQ instrument. [25]–[27] First, the number of participants that rated an item as ‘I cannot judge’ was calculated. Because of our heterogeneous study sample and the exploratory nature of the study, we applied a lenient cut-off of 33%; items that were rated by over 33% as ‘I cannot judge’ were excluded from further analysis. Second, the data were aggregated from clinical teacher to the teaching team level. Subsequently, the median, 20^th^ and 80^th^ percentile scores of all items were calculated to inspect for extreme floor or ceiling effects. Later, a data reduction technique known as principal component analysis (PCA) was performed, to extract the number of factors (composite scales) underlying the TeamQ items. The varimax rotation method was used to extract the factors. [28] We used the eigenvalue (>1) criterion to determine the number of factors to extract. We also checked the scree plot. The interpretation of the factors was led by the factor loadings (>0.40) and the meaningfulness of the factors in relation to the theory. When both were conflicting, theory was leading because of the exploratory nature of this study and the relative small sample size of our population. Third, the internal consistency reliability coefficient (Cronbach's alpha) of the composite scales extracted during the PCA was calculated. Cronbach's α of >0.70 was considered as reasonable reliability, α>0.80 was considered as good reliability. As an additional measure of the consistency and reliability of the scales, the corrected item-total scale correlation was calculated for each item. Subsequently, we checked for overlap between the scales by calculating the inter-scale correlations. Ideally, inter-scale correlations are below 0.70 (which corresponds to an overlap of <50%). Lastly, we correlated the scales with two, for this instrument developed, global items of teamwork: ‘How do you rate your own contribution to the teaching team's teamwork?’ and ‘How do you rate this team's teamwork?’[29] These correlations provided an indication of the construct validity of the composite scales and were expected to be in the range between 0.30 and 0.80 for an indication of good construct validity. Finally, we were interested in the number of clinical teacher evaluations needed to obtain reliable scale and total scores of teamwork in teaching teams. The number of evaluations was the only random variance component of interest, so in generalizability theory terminology we had a single-facet nested design. Because generalizability theory was designed for fully crossed designs (not for nested designs), with more than two random facets, more efficient alternatives to obtain the number needed for reliable scale and total scores are available for studies with a single-faceted nested design. [30] One of these alternatives is based on the assumption that the ratio of the sample size (N) to the reliability coefficient (R) would be approximately constant across combinations of sample size and associated reliability coefficients. [31] Therefore, R_new_ and N_new_ can be calculated from the already known R_old_ and N_old_ (as observed in this study) by the formula N_new_/R_new_  =  N_old_/R_old_. In previous studies, this method yielded similar results to the computationally exhausting generalizability analysis. [26], [32] In this study we calculated the number of evaluations needed to obtain the pre-defined α coefficients of 0.60, 0.70, 0.80 and 0.90 for the scales and the total score of the TeamQ. To triangulate this measure, we also calculated the observed α coefficients for residency training programs evaluated by 2 to 5, 6 to 9 and more than 9 team members. All analyses were performed using SPSS 20.0 for Windows.

To answer the second research question: how do individual clinical teachers evaluate their current levels of teamwork, we calculated the median score, 20^th^ and 80^th^ percentile score for all items. The clinical teachers all scored their self-reported performance of teamwork in a rating of a 5-point Likert scale ranging from ‘Very low degree of application’ to ‘Very high degree of application’.

## Results

### 1. Selecting items with Delphi

The Delphi expert group consisted of 5 clinical teachers and 13 program directors. These respondents have a mean (±SD) of 27 (±8) years clinical experience and 12 (±9) years of experience as a clinical teacher. Together with 7 residents, they represented the various surgical (12 respondents), medical (13) and auxiliary (1) specialties. In addition, 6 educationalists participated in the Delphi rounds as experts (Table 1). Based on the ratings of the 32 experts participating in the first Delphi round - evaluating relevance -, 26 out of the initial 86 items were excluded. In the second Delphi round, the remaining 60 items were reviewed by 25 experts for clarity and priority (Table 1). In addition, the research group discussed the results using the three theoretical teamwork lines (individual teamwork skills, team process and team results), and decided to exclude a further 6 items. Finally, 54 items remained in the preliminary TeamQ instrument to be pilot tested in practice.

**Table 1:** Characteristics of the participants in the modified Delphi procedure.

Number of participants	31
Number of males	19
Number of based at an academic teaching hospital	23
Number of program directors	13
Number of clinical teachers	5
Number of residents	7
Number of educational professionals	6

### 2. Testing TeamQ instrument

In total, 114 teaching teams with 929 clinical teachers (64%) used the TeamQ instrument in the pilot phase. Two teams were excluded from the analysis because only one team member responded. Team size varied from small (<10 team members; 42% of teams included in the study), to medium (10–20 team members; 46% of the teams included) to large groups (>20 team members; 12% of teams). Of all groups, 39% were teams from surgical specialties, 46% from non-surgical and 15% from auxiliary disciplines. Forty percent of all teams provided postgraduate medical training in University Medical Centers (40%) and 60% in teaching hospitals. The median response per team was 7, 20^th^ and 80^th^ percentile scores were 4 and 11 (Table 2).

**Table 2:** Characteristics of the participants in the testing phase of the TeamQ instrument.

Number of teaching teams	114
Number of clinical teachers who completed the TeamQ instrument (percentage of those invited)	929. (64%)
Median number of evaluations completed per teaching team (20^th^–80^th^ percentile)	7 (4–11)
Number of small sizedteams (<10 clinical teachers):	47
Number of medium sized teams (10–20 clinical teachers):	53
Number of size of large teams (>20 clinical teachers):	14
Number of surgical teaching teams^1^	44
Number of Non-surgical teaching teams 2	53
Number of auxiliary teaching teams^3^	17
Number of teaching teams based ad an university medical center	46

1 Obstetrics/gynaecology, Surgery, Ear, nose and Throat surgery, Neurosurgery, Ophthalmology, Orthopaedic surgery, Plastic and Reconstructive surgery, Thoracal surgery.

2 Dermatology, Internal Medicine, Pulmonology, Gastro-enterology, Neurology, Psychiatry, Rehabilitation Medicine, Cardiology, Paediatrics, Emergency Medicine.

3 Pharmacy, Anaesthesiology, Microbiology, Nuclear medicine,

Pathology, Radiology, Radiotherapy, Clinical Genetics.

### 3. Statistical analyses

Five items were rated as ‘I cannot judge’ by over a third (38% to 53%) of the clinical teachers. These items are listed in Table S1 and were removed before conducting the principal component analysis. Subsequently, principal component analysis (PCA) was performed on 49 items. The extraction of the items onto the composite TeamQ scales was based on factor loadings and the content of the items in relation to the theory of teamwork. Factor loadings of >0.40 on a composite scale were considered. When items had factor loadings of >0.40 on multiple scales, the items were placed in the scale where they fit best, based on 1) three theoretical research lines, [5]–[7] or 2) highest factor loading. We reflected within the research group on these three theoretical lines by deciding which scale the 10 items with a cross loading should be placed in. Consequently, the PCA revealed a 10-factor structure of the TeamQ questionnaire that explained 70% of the variance among teaching teams. However, based on discussion within the research group, it was decided to exclude two factors because they contained only 2 items. One item had low factor loadings on all remaining 8 factors and based on theory this item was not essential to retain in the TeamQ instrument; therefore, this item was excluded at this stage. The remaining 8 factors (that contained a total of 48 items) were labeled as *task expertise; team expertise; decision-making*; *team leadership*; *feedback culture; team results; engaging residents* and *residents' empowerment*. The eight scales of the TeamQ contained 3 to 11 items per scale. Factor loadings and corrected item-total scale correlations are presented in Table 3 and 4. The reliability of the TeamQ scales was ≥0.70 for seven scales, ranging from 0.75 for *decision-making* to 0.93 for *team leadership*. The scale for *residents' empowerment* had a reliability coefficient of 0.66.

**Table 3:** Median scores, factor loadings and corrected item-total scale correlations, for the TeamQ items.

		Median Scores (20^th^–80^th^ percentile score)	Factor loadings on primary scale	Corrected item – total scale correlations
**Theme**	**Task Expertise**	3.48 (3.07–3.77)		
TaE01	I take training courses to keep my teaching qualities up to scratch.	3.52 (3.00–4.00)	0.44	0.44
TaE02	I know exactly what is involved in ‘modernising the teaching program’.	3.42 (3.00–3.75)	0.71	0.66
TaE03	I can give examples of concrete improvements brought about by the modernisation of the teaching program.	3.28 (3.00–3.67)	0.79	0.60
TaE04	The local teaching plan is approved by all members of the teaching team.	3.82 (3.18–4.50)	0.64	0.50
TaE05	I understand the results of our teaching program.	3.12 (2.71–3.67)	0.56	0.57
**Theme**	**Team Expertise**	3.57 (3.07–3.99)		
TeE01	We make a joint decision on whether a resident can proceed to the next phase of his or her program.	4.00 (3.25–4.50)	0.75	0.70
TeE02	We discuss in the teaching team any differences of opinion about how the residents perform.	4.13 (3.60–4.43)	0.76	0.69
TeE03	We discuss in the teaching team any problems in how we work together.	3.33 (2.80–3.90)	0.53^1^	0.65
TeE04	I discuss with my colleague(s) my opinions about how we train residents.	3.57 (3.14–4.00)	0.72	0.72
TeE05	I discuss with my colleague(s) how we monitor the quality of our teaching.	3.33 (2.86–3.76)	0.46^2^	0.59
TeE06	I discuss with my colleague(s) how the teaching tasks are divided.	3.28 (2.89–3.83)	0.40	0.54
TeE07	I discuss with my colleague(s) my experiences with training residents.	3.67 (3.33–4.00)	0.69	0.69
**Theme**	**Team Decision-making**	3.82 (3.56–4.10)		
TD01	Our teaching meetings are very effective.	3.71 (3.40–4.00)	0.54	0.55
TD02	I can express my opinions honestly and openly.	4.00 (3.69–4.50)	0.44^3^	0.51
TD03	I understand the role and duties of the Program Director.	4.00 (3.69–4.37)	0.60	0.59
TD04	Our decision-making is in line with an agreed procedure.	3.25 (2.88–3.75)	0.37	0.44
TD05	I understand my duties as a clinical teacher.	4.00 (3.80–4.33)	0.34	0.54
**Theme**	**Program Directorship**	3.69 (3.31–4.02)		
TL01	I can approach the Program Director if I need help with teaching activities.	4.00 (3.50–4.40)	0.79	0.78
TL02	The Program Director encourages me to do my best for the teaching program.	3.67 (3.21–4.00)	0.79	0.74
TL03	The Program Director has put ‘the vision for teaching’ on the agenda in the past year when discussing teaching issues.	3.11 (2.54–3.60)	0.71	0.68
TL04	The Program Director inspires me and my colleagues to carry out our work on the basis of a shared vision of teaching.	3.38 (2.90–3.83)	0.83	0.84
TL05	The Program Director invites me and my colleagues to exert our influence on teaching issues.	3.59 (3.13–4.00)	0.76	0.77
TL06	The Program Director encourages me and my colleagues to train residents in line with the teaching plans.	3.62 (3.09–4.00)	0.77	0.78
TL07	The Program Director ensures there is a careful decision-making procedure in the teaching team when discussing the level of performance of the residents.	4.00 (3.60–4.33)	0.61	0.57
TL08	The Program Director regularly talks to the residents about their performance.	4.32 (4.00–4.60)	0.76	0.69
TL09	The Program Director regularly informs the teaching team of the decisions of the CTC (Central Teaching Committee) of the hospital.	3.33 (2.83–3.80)	0.56	0.60
TL10	I entrust the organisation of teaching activities to the Program Director.	4.48 (4.00–4.75)	0.80	0.73
TL11	The Program Director regularly discusses teamwork with the teaching group.	3.33 (2.98–3.92)	0.68	0.74
**Theme**	**Feedback Culture**	2.80 (2.37–3.12)		
FC01	I actively ask residents for feedback on how I perform as a teacher.	3.00 (2.50–3.50)	0.59^4^	0.57
FC02	I regularly reflect on my behaviour as a teacher.	3.18 (2.85–3.50)	0.56	0.63
FC03	In receive regular feedback from my colleague(s) on my performance as a teacher.	2.50 (2.00–3.00)	0.79	0.77
FC04	I regularly give my colleague(s) feedback on their performance as teachers.	2.44 (2.00–2.90)	0.76	0.81
FC05	I receive feedback from the Program Director/my colleagues on how I perform as a teacher.	2.63 (2.00–3.20)	0.52^5^	0.59
FC06	I always hold my colleague(s) to account for any unprofessional behaviour.	3.17 (2.71–3.60)	0.56	0.41
FC07	We discuss our personal areas for improvement in teaching in the teaching team.	2.60 (2.10–3.33)	0.54^6,7^	0.65
**Theme**	**Team Results**	3.64 (3.36–3.94)		
TR01	I observe that my fellow teachers all make an equal contribution to achieving our teaching goals.	3.40 (3.00–3.71)	0.27	0.47
TR02	I have a clear picture of what we as a teaching team want to have achieved in five years' time in terms of our teaching.	3.35 (3.00–3.80)	0.45^8^	0.60
TR03	I am aware that the way we work together within our teaching team is an example to the residents.	4.00 (3.60–4.50)	0.72	0.67
TR04	There is consensus within our teaching team about the medical policies to be applied.	3.89 (3.67–4.09)	0.71	0.50
TR05	I agree with the way we divide the teaching tasks among our team members.	3.67 (3.25–4.20)	0.52^9^	0.60
TR06	We have made clear agreements about our teaching activities.	3.60 (3.25–4.00)	0.44	0.53
**Theme**	**Engaging residents**	3.44 (3.10–3.85)		
REn01	In supervising residents, I always follow the residents' individual teaching plans.	2.90 (2.50–3.33)	0.64	0.48
REn02	If a resident needs a specific type of supervision and one of my colleagues is more skilled at this than me, I would refer the resident to my colleague.	3.46 (3.00–4.00)	0.82	0.71
REn03	If a resident wants to learn specific aspects of patient care with which one of my colleagues has more experience, I will refer the resident to this colleague.	4.00 (3.60–4.33)	0.71^10^	0.61
REn04	If I need help, I ask my colleague(s) for support in carrying out teaching tasks.	3.50 (3.14–4.00)	0.57	0.54
**Theme**	**Residents**' **Empowerment**	4.00 (3.72–4.17)		
REm01	I expect residents to take responsibility for their own education.	3.84 (3.60–4.17)	0.57	0.51
REm02	I am aware of residents' capabilities, so I am able to supervise them effectively.	3.81 (3.50–4.00)	0.30	0.44
REm03	I value the residents' contribution to the teaching meetings.	4.27 (4.00–4.50)	0.71	0.47

Cross loading(s) (≥0.40) of the item(s) scale (factor loading): 1 =  Team result(0.48), 2 =  Feedback culture (0.40), 3 =  Team result (0.40), 4 =  Engaging residents (0.50), 5 =  Program Directorship (0.53), 6 =  Team expertise (0.40), 7 =  Team result (0.48), 8 =  Feedback culture(0.40), 9 =  Decision-making (0.45), 10 =  Resident's Empowerment(0.42).

**Table 4:** Internal consistency reliability coefficients (Cronbach's α) for all themes of the TeamQ instrument.

Theme	Cronbach's α
Task Expertise	0.77
Team Expertise	0.87
Team Decision-making	0.75
Program Directorship	0.93
Feedback Culture	0.84
Team Results	0.80
Engaging residents	0.77
Residents' Empowerment	0.66
All TeamQ items combined	0.96

The inter-scale correlations revealed satisfactory overlap between the scales (all ≤0.71, Table 4). The correlations between the scales and ‘global item 1’: “How do you rate your own contribution to the teaching teams' teamwork?” were within the expected range (0.30–0.80) for seven scales; however, the correlation was lower for the *team leadership* scale (0.23). The correlations between ‘global item 2’: “How do you rate this team's teamwork?” and the scales were all within the expected range specified above (Table 5).

**Table 5:** Inter-scale and scale – global item correlations of the TeamQ themes (Pearsons' correlation coefficients).

	Task expertise	Team expertise	Decision-making	Program Directorship	Feedback culture	Team result	Engaging residents	Residents' empowerment
Team expertise	0.38	1	-	-	-	-	-	-
Decision-making	0.48	0.69	1	-	-	-	-	-
Program Directorship	0.43	0.40	0.56	1	-	-	-	-
Feedback culture	0.50	0.64	0.57	0.44	1	-	-	-
Team result	0.49	0.68	0.71	0.46	0.61	1	-	-
Engaging Residents	0.30	0.46	0.35	0.28	0.50	0.46	1	-
Residents'empowerment	0.22*	0.53	0.56	0.28	0.44	0.50	0.55	1
Global 1: How do you rate your own contribution to the teaching team's teamwork?	0.40	0.54	0.47	0.23*	0.55	0.64	0.30	0.44
Global 2 How do you rate this team's teamwork?	0.40	0.60	0.50	0.31	0.44	0.72	0.36	0.36

*p<0.05 (all other correlations had p<0.01).

**Figure 1:**
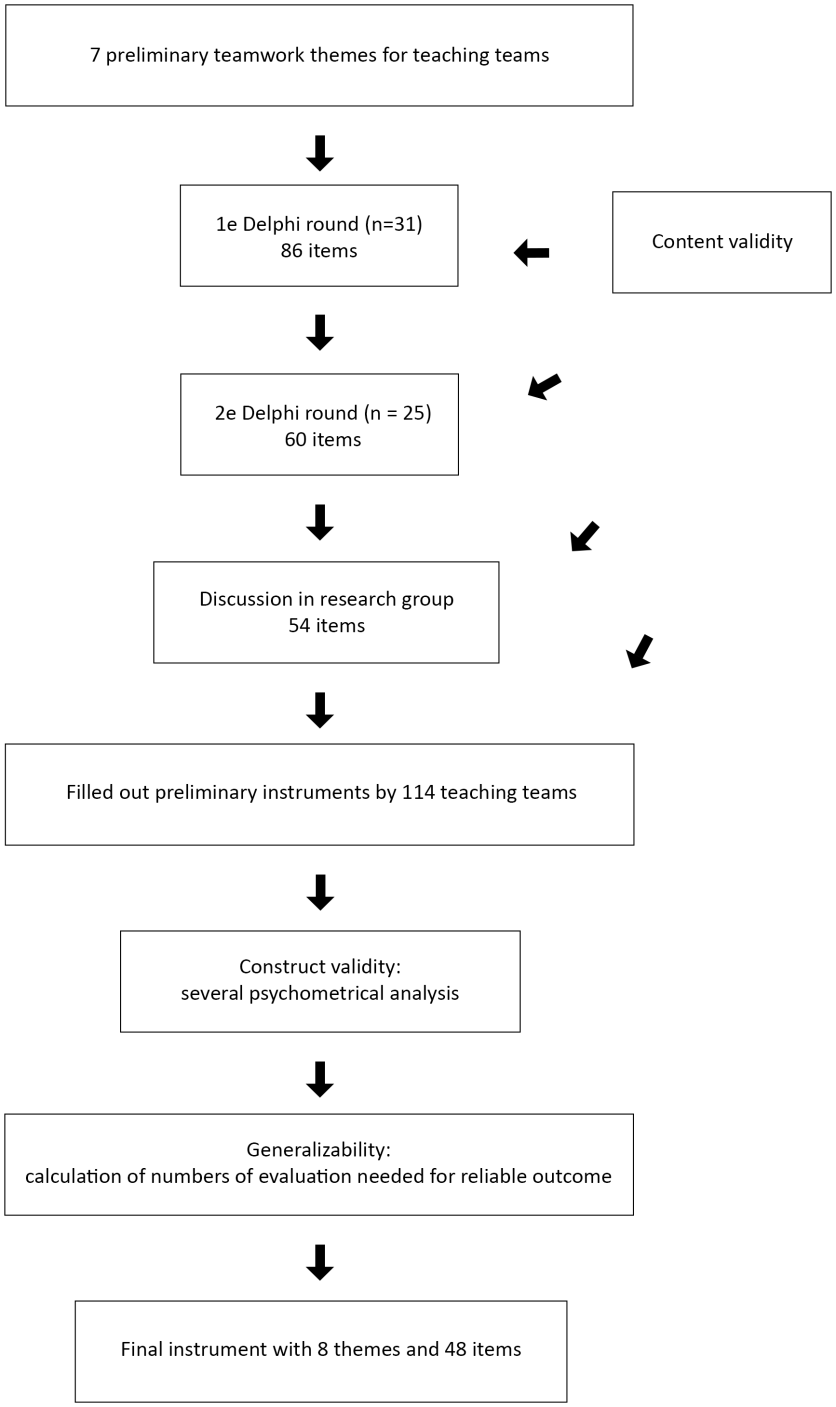
Flowchart of different steps in developing and validating TeamQ measurement instrument.

The generalizability analysis based on the formula presented in the methods section revealed that 5 to 6 completed evaluations were needed to obtain reliability coefficients for the scale of 0.60, 5 to 7 evaluations were needed for a coefficient of 0.70, 6 to 8 evaluations were needed for a coefficient of 0.80 and 7 to 10 evaluations were needed for a coefficient of 0.90. The smallest number of evaluations were needed to obtain reliable measures for the *team leadership* scale and the greatest number were needed to obtain reliable measures for the *residents' empowerment* scale (Table 6). The observed reliability measures of the TeamQ scales for teaching teams that completed 2 to 5 evaluations ranged from 0.69 for *decision-making* to 0.93 for *team leadership*. The reliability for teams that completed 6 to 9 or 10 or more evaluations was >0.72 for seven scales; only the *resident empowerment* scale had low reliability levels (0.53 and 0.39 respectively) (Table 7). Figure 1 visualizes all the different steps in developing and validating TeamQ questionnaire.

**Table 6:** Number of completed TeamQ evaluations needed to obtain reliable theme scores, based on generalizability analysis.

Theme	Reliability coefficient (α) of 0.60	Reliability coefficient (α) of 0.70	Reliability coefficient (α) of 0.80	Reliability coefficient (α) of 0.90
Task expertise	5	6	7	8
Team expertise	5	6	6	7
Decision-making	6	7	7	8
Program Directorship	5	5	6	7
Feedback culture	5	6	7	8
Team result	5	6	7	8
Engaging Residents	5	6	7	8
Residents' empowerment	6	7	8	10
All TeamQ items combined	4	5	6	7

**Table 7:** Observed reliability levels (α) for teams with a different number of completed TeamQ evaluations.

Theme	2 to 5 evaluations	6 to 9 evaluations	10 or more evaluations
Number of teams	N = 44	N = 32	N = 38
Task expertise	0.78	0.76	0.72
Team expertise	0.86	0.88	0.85
Decision-making	0.69	0.78	0.80
Leadership	0.93	0.93	0.93
Feedback culture	0.84	0.89	0.87
Team result	0.77	0.80	0.84
Engaging residents	0.72	0.76	0.80
Residents'empowerment	0.71	0.53	0.39
All TeamQ items combined	0.94	0.96	0.96

### 4. Evaluating teamwork

Clinical teachers gave the highest median scores to the teamwork theme of *residents' empowerment* (4.00). The scale with the lowest median score was *feedback culture* (2.80). The other teamwork themes were all rated between 3.44 and 3.82, namely: *task expertise* (3.48)*; team expertise* (3.57); *decision-making* (3.82); *team leadership* (3.69); *team results* (3.64); *engaging residents* (3.44) (Table 3).

## Discussion

This study reported how the TeamQ instrument was developed in a three-step process, resulting in a practice and theory-based, rigorously tested instrument. From the 54 initial items which were piloted in 114 teams, 48 are now included in the final TeamQ instrument and can be used for valid and reliable measurement of teamwork in teaching teams. Further, we found that clinical teachers in general positively evaluate their teamwork. The teams' feedback culture left most room for improvement. We will now discuss the answers to our two research questions by reflecting on the findings presented. We will start with discussing the results of the validation process, using the standard development and validation criteria: content validity, construct validity and internal consistency. [33]

First, a comprehensive and thorough analysis was conducted of the content validity of this study. Since we aimed for developing an theoretically founded instrument that was specifically fit for clinical teachers, we build on theory on teamwork and the preliminary themes and quotes from a previous focus group study of teamwork in teaching teams. [4] The relevance of the preliminary items for teamwork in teaching teams was tested in a Delphi round by 31 experts. A significant number of items were excluded in this Delphi round based on limited relevance. All remaining items were rated by the experts as very relevant for evaluating teamwork in teaching teams. This contributed to the content validity of the items that were tested among 114 teaching teams in this study. The second validity criterion evaluated in this study was the construct validity. The psychometric analyses of this study revealed that the items cluster together in an 8-factor structure. The explained variance of the factors, the desirable correlations between the themes and the desirable correlations of the themes with the two global items of teamwork all contributed to the construct validity of the TeamQ.

We found some differences between the preliminary 7-theme structure that was based on our previous focus group study and the current 8 themes that were identified based on the psychometric analysis. This is a natural result of this exploratory phase in the validation process. The analysis presented in this study represents the first quantitative test of the preliminary structure that was based on a qualitative exploration. At that stage changes and refinement are expected and desired, while at a later stage when confirmatory techniques will be used, changes are undesirable.

The third validity criterion is evaluated the internal consistency reliability. The reliability of the TeamQ scales was found to be adequate for seven out of the eight scales, with *team leadership* exhibiting the highest reliability and *decision-making* the lowest. TeamQ can therefore be considered a feasible instrument for measuring teamwork in teaching teams. The *residents' empowerment* scale had a low reliability coefficient of 0.66. The scale contains only three items, as does the *engaging residents* scale. Having a team result that focuses clearly on the residents can be an important impetus for teamwork in teaching teams. However, as known from the literature, the result of teamwork is not always sharply defined in the minds of the team members. [15] It may be necessary to employ a qualitative research method to explore in greater depth these two scales that represent the result of teamwork in teaching teams for residents.

### The current level of teamwork

We also explored the research question: how individual members of teaching teams appraise their current levels of teamwork. This study shows that in general, clinical teachers evaluate their current level of teamwork positively. This study shows that clinical teachers report that their current teamwork situations are to a large extent congruent with the ideally phrased teamwork statements in the questionnaire. This suggests that they evaluate their current levels of teamwork positively. The highest and lowest scoring teamwork scales are *residents' empowerment* and *feedback culture*. The high score on *residents' empowerment* may possibly be attributable to the fact that clinical teachers, although they do not see this as a result of teamwork, are nonetheless focused on the residents in their role as clinical teachers. The low scores on *feedback culture* indicate the problems with feedback in teamwork of teaching teams. This is in line with another study which also reported that giving and receiving feedback is a difficult skill to master. [34] Different organizational studies endorse feedback as a key element of teamwork. [35], [36] Through feedback, a team can obtain information about the quality and quantity of its output as well as knowledge about the effectiveness of the method used to achieve the desired levels of performance. Feedback in teamwork serves as an error detection device and thus as a stimulus to begin to identify and resolve problems. [35] We suggest that, if clinical teachers develop the teamwork skills of giving and receiving feedback, the quality of assessment and supervision of the residents may improve. It may also have a positive effect on the quality of teamwork between clinical teachers in postgraduate medical training programs. [15]

### Strengths and Limitations

We consider the combination of theory and practice and the use of both qualitative and quantitative methods in developing the TeamQ instrument as strengths of this study. The multi-center and multi-specialty character of the sample and the high response rate of the TeamQ questionnaires are also strong points. The strength of the Delphi procedure lies in the diversity of the four expert groups and the role of the research group in the modified procedure. The testing of the preliminary instrument was successful because the instrument was readily available and interested teaching teams had easy access to it. Given these strengths, we regard TeamQ as a valuable instrument for evaluating teamwork in teaching teams. However, validation must be seen as a continuous process. This study's sample did not allow for subgroup analysis, which may be considered a limitation of the study; it limits our knowledge of the applicability of TeamQ for specific situations that may benefit from more detailed analysis. Such situations could include, for example, the reliability for large and small sized groups, for different specialties and different settings. A larger sample would allow subgroup analysis in future research.

### Implications for Clinical Education, Research and Policy

Teaching teams could evaluate teamwork regularly as part of continuous improvement of the quality of post-graduate medical education. [15], [29] In particular, teamwork evaluations might be useful when major changes in teams occur, such as changes in team composition, or when teams are presented with major challenges, such as accreditation of residency training. Teamwork evaluations may be performed to comply with accountability requirements. In order to improve teamwork it is important to know the strengths and weaknesses of working together, but solely measuring teamwork in teaching teams does not necessarily lead to improvement. Successful implementation processes within healthcare have shown the importance of taking into account clinical teachers' readiness to change. [15], [34] To improve their teamwork, clinical teachers need to devote time and attention to working on the required improvements, as well as the willingness to change. Once the TeamQ evaluation has been carried out, team coaching and training can be introduced to further develop individual teamwork skills. [10], [37], [38] Future TeamQ research should include continuous validation of the instrument to monitor and further improve the quality of the instrument and to adjust to changes in teamwork in the context of post graduate medical education. In line of this explorative validation, future research can expand evidence about convergent, predictive and concurrent validity of the TeamQ instrument. For example the TeamQ scores can be related to other quality measurement instrument and in other contexts, i.e. in different geographical, cultural and health care systems contexts.

## Conclusions

This study provides a first indication of the validity and reliability of a new instrument for measuring teamwork in teaching teams in post-graduate medical training. The TeamQ instrument is now available and has been found to be reliable for use by both small and large teaching teams. The high response rates and the limited number of evaluations needed for reliably measuring teamwork indicate the feasibility of the TeamQ instrument in the evaluation of teamwork in teaching teams in practice. The use of TeamQ may the first step in an improvement process; indeed the TeamQ results need to be followed up by reflection and an action plan to achieve real improvement. Clinical teachers are least positive about the feedback culture in their teaching team. Facilitating the further development of individual teamwork skills, i.e. training and coaching in receiving and giving feedback, may be instrumental in realizing positive change. [15], [34]

## Supporting Information

Table S1 **Characteristics of themes and items preliminary instrument.**
(DOC)

Document S1 **Letter of approval of the Institutional Ethical Review Board of the Academic Medical Center, Amsterdam.**
(PDF)
