# Supplementary material for: Development and Validation of an Instrument for Measuring the Quality of Teamwork in Teaching Teams in Postgraduate Medical Training (TeamQ)
Source: PLoS One. 2014 Nov 13;9(11):e112805. doi: 10.1371/journal.pone.0112805 (PMC4231160; doi:10.1371/journal.pone.0112805)
Supplement: Table S1 — Characteristics of themes and items preliminary instrument. (DOC) [file pone.0112805.s001.doc]

**Table S1:** Characteristics of themes and items preliminary instrument

| 1. **The clinical teacher in a teaching team: who are we? what do we do?** | Excluded due to relevancy:  Delphi 1 | Excluded due to clarity & priority: Delphi 2 | Excluded due to statistics | Included in the final TeamQ instrument |
| --- | --- | --- | --- | --- |
| 1. I feel that I am a clinical teacher 24 hours a day. | x |  |  |  |
| 1. I am an expert at my job in terms of clinical teaching. |  |  | All factor loadings < 0.19 |  |
| 1. I know my job as a clinical teacher. |  |  |  | TD05 |
| 1. With our passion for our work and our patients, we stimulate the residents’ passion for the specialist field. |  | x |  |  |
| 1. As a teaching team, we are aware of the importance of teaching as a means to safeguard the quality of our specialist field. |  | x |  |  |
| 1. I follow training courses to keep my teaching skills up to scratch. |  | R* |  | TaE01 |
| 1. I play an active role in the clinical teaching team. |  | x |  |  |
| 1. I have a clear idea of what we as a teaching team want to have achieved in five years in the area of teaching. |  | R |  | TR02 |
| 1. There is consensus within our teaching team about the medical policies to be applied. |  | R |  | TR04 |
| 1. I am aware that the way we work together within our teaching team is an example to the residents. |  | R |  | TR03 |
| 1. As members of the teaching team we strive for maximum diversity. | x |  |  |  |
| 1. I am happy to be a member of the teaching team. | x |  |  |  |
| 1. I am sure that my fellow teachers all make an equal contribution to achieving our teaching goals. |  | R |  | TR01 |
| 1. I recognise similarities between my fellow teachers and myself. | x |  |  |  |
| 1. I am convinced that teachers can perform better in the area of teaching if they work together rather than alone. |  | x |  |  |
| 1. I am happy with our team performance in the area of clinical teaching. | x |  |  |  |
| 1. We are accountable for the quality of our activities. |  | x |  |  |
| 1. I have all the information I need and in good time to be able to supervise the residents. |  | x |  |  |
| 1. We discuss our personal areas for improvement within the teaching team. |  |  | R | TC07 |
| 1. I strive to maintain my skills in the area of patient care. |  | x |  |  |
| 1. I am aware that the way we work together contributes to better training for the residents. |  | x |  |  |
| **2) The residents: what’s our aim?** |  |  |  |  |
| 1. We monitor our shared ideas about how residents learn from the different clinical teachers in the teaching team. | x |  |  |  |
| 1. Our teaching enjoys a good reputation among the residents in our region. | x |  |  |  |
| 1. I do my best to ensure I have a good personal relationship with the residents. | x |  |  |  |
| 1. I am aware of the residents’ capabilities, so I am able to supervise them effectively. |  | R |  | REm02 |
| 1. We demonstrate diverse ways of working so that the residents can learn from the differences. | x |  |  |  |
| 1. I expect residents to take responsibility for their education. |  | R |  | REm01 |
| 1. We involve the residents when recruiting new colleagues, because we want them to be an integral part the group. |  | x |  |  |
| 1. We strive for a stratified group of residents in terms of culture, sex and stage of the program, so that they can learn optimally from one another. | x |  |  |  |
| 1. I actively ask the residents for feedback on my performance. |  | R |  | TC01 |
| 1. We safeguard the professional relationship between clinical teachers and residents. |  |  |  |  |
| 1. We apply a single professional standard with regard to the residents. | x |  |  |  |
| **3) The team leader as Program Director: what does the leader do?** |  |  |  |  |
| 1. I know what to expect from the Program Director. |  | x |  |  |
| 1. I have a clear understanding of the interplay of different influences and the position, knowledge and experience on which the Program Director bases his leadership. | x |  |  |  |
| 1. I can ask the Program Director for advice on clinical teaching activities. |  | R |  | TL01 |
| 1. The Program Director inspires me and my colleagues to work from a shared vision about clinical teaching. |  | R |  | TL04 |
| 1. The Program Director discusses teaching issues with the residents and members of the teaching team, the members of the hospital board and within the academic association. |  | R: splitting in 4 items | 2 items not to judge | TL08 & TL09 |
| 1. The Program Director invites me and my colleagues to have an influence on teaching issues. |  | R |  | TL05 |
| 1. I receive feedback from the Program Director on my performance as a clinical teacher. |  | R |  | TC05 |
| 1. The Program Director makes sure there is continual awareness and continual innovation in the teaching. |  | x |  |  |
| 1. The Program Director ensures there is a meticulous decision-making procedure in place within the teaching team when the level of performance of the residents is under discussion. |  | R |  | TL07 |
| 1. The Program Director encourages me to do the best I can in my clinical teaching. |  | R |  | TL02 |
| 1. The Program Director emanates leadership qualities. |  | x |  |  |
| 1. I entrust the organisation of the teaching activities to the Program Director. |  |  |  | TL10 |
| 1. The Program Director takes responsibility for the quality of the teaching, on behalf of the teaching team. |  |  | x= not to judge |  |
| 1. The Program Director regularly talks to the teaching team about their mutual cooperation. |  | R |  | TL11 |
| **4) The issues that we share: what are these issues?** |  |  |  |  |
| 1. I discuss with my colleagues my opinion about how we train residents. |  | R |  | TeE 04 |
| 1. The local teaching plan has been endorsed by all members of the teaching team. |  | R |  | TaE 04 & TaE05 |
| 1. We base our discussions with residents on the post-graduate medical program. | x |  |  |  |
| 1. If I am unable to complete my clinical teaching duties, I will say so will help seek a solution. | x |  |  |  |
| 1. I discuss with my colleagues how we can safeguard the quality of our clinical teaching. |  | R |  | TeE05 |
| 1. I discuss the division of clinical teaching tasks with my colleagues. |  | R |  | TeE06 |
| 1. I am aware of the ambitions of my fellow clinical teachers. | x |  |  |  |
| 1. We take decisions jointly about how we develop our cooperation as a teaching team. | x |  |  |  |
| 1. We discuss decisions about the teaching plans and the assessment of the residents, before making decisions. |  | x |  |  |
| 1. I discuss any problems I have with carrying out the clinical teaching tasks. | x |  |  |  |
| 1. I discuss with my colleagues my experiences with teaching residents. |  | R |  | TeE07 |
| 1. We decide together whether a resident should proceed to the next stage of his or her program. |  |  |  | TeE01 |
| 1. I share my thoughts about the training of residents. | x |  |  |  |
| **5) The teamwork structure: how are we organised?** |  |  |  |  |
| 1. I am happy with the way we have organised the clinical teaching. | x |  |  |  |
| 1. In supervising residents, I always adhere to the residents’ individual teaching plans. |  | R |  | REn01 |
| 1. I cooperate in safeguarding an adequate decision-making procedure, and ensure decisions are handled properly. | x |  |  |  |
| 1. Our teaching meetings are efficient. |  |  | R | TD01 |
| 1. I agree with the way the clinical teaching activities are divided among the team. |  | R |  | TR05 |
| 1. We regularly take time out with the teaching team to evaluate and formulate our teaching policy. | x |  |  |  |
| **6) The feedback culture: how do we behave towards one another?** |  |  |  |  |
| 1. If a fellow teacher behaves in an unprofessional way, I will always hold him or her to account for this. |  | R |  | FC06 |
| 1. If there are problems with our teamwork, we discuss the issues in our teaching team. |  | R |  | TeE03 |
| 1. We hold one another to account if anyone pays lip service to the fact that clinical teaching is important, but does not put this into practice. |  | x |  |  |
| 1. I do not avoid conflicts and am therefore able to resolve problems. | x |  |  |  |
| 1. We discuss in our teaching team the areas where we personally need to improve our clinical teaching. |  | R |  | FC07 |
| 1. I regularly reflect on my performance as a clinical teacher. |  | R |  | FC02 |
| 1. We have a structured program to give one another feedback on how we perform as clinical teachers. |  | x |  |  |
| 1. Because we hold one another to account on how we perform in our clinical teaching, we are able to promote teamwork. | x |  |  |  |
| 1. I compliment my colleagues on how they perform as clinical teachers. |  | x |  |  |
| 1. We use conflicts to learn from them, rather than avoiding them. | x |  |  |  |
| 1. We strive for a culture in which teachers consider it logical to receive feedback and to be assessed. |  | x |  |  |
| 1. We hold everyone accountable for our mutual commitment. | x |  |  |  |
| 1. We are prepared to discuss our own behaviour within the clinical teaching team. |  | x |  |  |
| **7) The environment: what is happening around us?** |  |  |  |  |
| 1. I know exactly what is involved in modernising post-graduate medical training. |  | R |  | TaE02 |
| 1. The assessment visitation has made us aware of the importance of the quality of our clinical teaching. | x |  |  |  |
| 1. In this hospital we all receive a teaching allowance to emphasise the team feeling. | x |  |  |  |
| 1. We understand the quality of the clinical teaching within the team. |  | x |  |  |
| 1. We work within the region with a regional plan, to which we have committed. | x |  | x = not to judge |  |
| 1. We are regarded as good clinical teachers by our scientific association. | x |  |  |  |
| 1. I take seriously the teaching innovations initiated by the scientific associations. | x |  |  |  |
| 1. We follow national and international developments in the field of clinical teaching. | x |  |  |  |

*R=Reformulate
