# Supplementary material for: Development and Validation of an Instrument for Measuring the Quality of Teamwork in Teaching Teams in Postgraduate Medical Training (TeamQ)
Source: PLoS One. 2014 Nov 13;9(11):e112805. doi: 10.1371/journal.pone.0112805 (PMC4231160; doi:10.1371/journal.pone.0112805)
Supplement: Document S1 — Letter of approval of the Institutional Ethical Review Board of the Academic Medical Center, Amsterdam. (PDF) [file pone.0112805.s002.pdf]

Academisch Medisch Centrum
Universiteit van Amsterdam

Aan de heer prof.dr. M.J. Heineman
Gynaecologie en Obstetrie
H4-220

Medisch Ethische Toetsingscommissie
E2-236
doorkiesnummer: 566 7389/566 6730
fax: 5669015

Amsterdam,16 april 2012
uw kenmerk:
ons kenmerk: W12_073 # 12.17.0090
betreft:
Uw brief: Onderzoek naar het functioneren van medisch specialisten in hun rol van opleiders van arts-assistenten in opleiding tot specialist (aios)

Geachte heer Heineman,

Uw brief d.d. 8 maart 2012 betreffende bovengenoemde studie is op 27 maart jl. besproken in de vergadering van het dagelijks bestuur.

Naar ons oordeel valt bovengenoemde studie niet binnen de reikwijdte van de wet medisch-wetenschappelijk onderzoek met mensen daar er bij deze studie geen sprake is van een medisch wetenschappelijke vraagstelling.

Derhalve behoeft het voorstel formeel niet te worden beoordeeld door een erkende toetsingscommissie.

Met vriendelijke groet,
namens de Medisch Ethische Toetsingscommissie,

Mw.mr. A. Bosma
ambtelijk secretaris

Bijlage: verklaring in het Engels (z.o.z)

c.c. pdf per email: r.m.vanderleeuw@amc.uva.nl; m.j.lombarts@amc.uva.nl

To whom it may concern,

Referring to our letter of April 16, 2012 (reference number W12_073 # 12.17.0090) we are pleased to confirm that the Medical Research Involving Human Subjects Act (WMO) does not apply to the above mentioned study and that an official approval of this study by our committee is not required.

Yours sincerely,
on behalf of the Medical Ethics Review Committee,

Mrs. A. Bosma, LLM
secretary
